# Zero-valent iron sand filtration reduces concentrations of virus-like particles and modifies virome community composition in reclaimed water used for agricultural irrigation

**DOI:** 10.1186/s13104-019-4251-y

**Published:** 2019-04-11

**Authors:** Jessica Chopyk, Prachi Kulkarni, Daniel J. Nasko, Rhodel Bradshaw, Kalmia E. Kniel, Pei Chiu, Manan Sharma, Amy R. Sapkota

**Affiliations:** 1 Maryland Institute for Applied Environmental Health, University of Maryland School of Public Health, School of Public Health Building (255), 4200 Valley Drive, Room 2234P, College Park, MD 20742 USA; 2 Center for Bioinformatics and Computational Biology, University of Maryland, College Park, MD USA; 3 United States Department of Agriculture, Agricultural Research Service, Environmental and Microbial Food Safety Laboratory, Beltsville, MD 20705 USA; 4 Department of Animal and Food Sciences, University of Delaware, Newark, DE 19716 USA; 5 Department of Civil and Environmental Engineering, University of Delaware, Newark, DE 19716 USA

**Keywords:** Viral metagenomics, Virome, Epifluorescent microscopy, Reclaimed water, Antibiotic resistance, Zero-valent iron, Sand filtration

## Abstract

**Objective:**

Zero-valent iron sand filtration can remove multiple contaminants, including some types of pathogenic bacteria, from contaminated water. However, its efficacy at removing complex viral populations, such as those found in reclaimed water used for agricultural irrigation, has not been fully evaluated. Therefore, this study utilized metagenomic sequencing and epifluorescent microscopy to enumerate and characterize viral populations found in reclaimed water and zero-valent iron-sand filtered reclaimed water sampled three times during a larger greenhouse study.

**Results:**

Zero-valent iron-sand filtered reclaimed water samples had significantly less virus-like particles than reclaimed water samples at all collection dates, with the reclaimed water averaging between 10^8^ and 10^9^ and the zero-valent iron-sand filtered reclaimed water averaging between 10^6^ and 10^7^ virus-like particles per mL. In addition, for both sample types, viral metagenomes (viromes) were dominated by bacteriophages of the order *Caudovirales*, largely *Siphoviridae*, and genes related to DNA metabolism. However, the proportion of sequences homologous to bacteria, as well as the abundance of genes possibly originating from a bacterial host, was higher in the viromes of zero-valent iron-sand filtered reclaimed water samples. Overall, zero-valent iron-sand filtered reclaimed water had a lower total concentration of virus-like particles and a different virome community composition compared to unfiltered reclaimed water.

**Electronic supplementary material:**

The online version of this article (10.1186/s13104-019-4251-y) contains supplementary material, which is available to authorized users.

## Introduction

The intense use of groundwater for agricultural and other activities has led to substantial aquifer depletions globally [1, 2]. Consequently, demand has grown for technologies, such as zero-valent iron (ZVI) sand filters, to treat alternative irrigation water sources and allow for their use. These filters—initially designed to remove chlorinated compounds from groundwater—are composed of mixtures of sand and zero-valent iron [3]. Currently, they are being developed to remove multiple contaminants, including microorganisms, from diverse water sources [4–10]. Specifically, ZVI reduces *Escherichia coli* levels in water [4, 5], and can reduce titers of multiple viruses, including Murine norovirus, and several bacteriophage species [6, 7, 11]. However, ZVI has not been evaluated on its ability to remove complex viral populations, such as those within reclaimed water used for agricultural irrigation.

In reclaimed water, virus-like particles (VLPs) are estimated to be 1000-fold higher than in potable water, with the majority of viruses homologous to bacteriophages [12]. Bacteriophages are among the most abundant biological entities on earth and are critical components in food web-dynamics and nutrient cycling [13]. Moreover, phages can influence its bacterial host’s phenotype through the horizontal transfer of genes [e.g. antibiotic resistance genes (ARGs)] [14–17]. This is potentially of concern for wastewater treatment plants, which are reported to be reservoirs for ARGs [18]. To address the data gaps noted above, this study evaluated DNA viruses in reclaimed water and ZVI-sand filtered reclaimed water collected during a larger study [19].

## Main text

### Methods

#### Sample collection

Samples were collected during a larger greenhouse study that evaluated impacts of ZVI-sand filtration on antimicrobials, *E. coli* and total bacterial communities in reclaimed water used to irrigate lettuce [19]. Briefly, in Summer 2016, 240 L of chlorinated effluent was collected biweekly from a tertiary wastewater treatment plant in the Mid-Atlantic U.S. that employed grinding, activated sludge processing, and secondary clarification, and then stored treated effluent in an open-air lagoon where it was chlorinated prior to land application. After collection, water was delivered to the greenhouse and stored in 189 L storage barrels (Algreen Products Inc., Ontario, Canada).

#### ZVI-sand filter and filtration process

A commercially-available biosand filter (HydrAid®BioSand Water Filter, NativeEnergy, Burlington, VT, USA) was modified for use in this experiment [19]. Briefly, the filter vessel is made of opaque plastic and has a total volume of ~ 55.5 L. Fine filtration sand, provided with the filter [20, 21] and ZVI particles (Peerless Metal Powders and Abrasives Company, Detroit, MI) were sieved (particle size range of 400 µm to 625 µm) and mixed, generating a 50:50 volume/volume mixture. The porosity of the filter was approximately 0.52 [22], the average volumetric flow rate was ~ 5.6 L/min, the filtration rate was 18 L/min/m^2^ and the approximate ZVI contact time was 2.58 min [19].

To mimic the use of sand filters in agricultural settings where irrigation water is not needed every day due to periodic rain events, reclaimed water was filtered every five days. During each filtration event, a 20 L composite of the stored water was gravity filtered to accommodate the irrigation needs of the greenhouse study. To maintain the filter between filtration events, it was kept submerged in reclaimed water, and right before filtration, the five-day old water within the filter was completely flushed out by pouring 20 L reclaimed water through the filter and discarding it. A new 20 L composite reclaimed water sample was then filtered. From the total ~ 20 L ZVI filtrate, a 1 L subsample of filtrate (ZW sample) was collected, along with 1 L of unfiltered reclaimed water (RW sample), once a month for the present study on 6/21/2016, 7/30/2016, and 8/9/2016.

#### VLP quantification

Viral enumeration was performed using the “filter mount method” [23]. Briefly, aliquots (1 μL for RW and 100 μL for ZW) of formalin fixed samples were suspended in sterile deionized water (total volume of 1000 μL), vacuum filtered onto a 13 mm 0.02-µm Anodisc filter (Whatman, USA), and stained with SYBR Gold (Thermo Fisher Scientific, USA). Triplicate slides for each sample were made and counted within 24 h with an Olympus BX61 microscope (20 fields, 1000× magnification). VLPs were quantified with iVision software and differences in VLP counts between samples types were tested using a paired *t*-test with Bonferroni correction.

#### Virome preparation and sequencing

Each sample (1 L) was vacuum filtered through a 0.2 μm membrane filter (Pall Corporation, Port Washington, NY) to remove the cellular fraction and collected in sterile receiving flasks. Viral particles were then concentrated using 100 μL of a Fe solution (4.83 g FeCl_3_ into 100 mL H_2_O) and 1 mL of a resuspension buffer (0.1 M EDTA-0.2 M MgCl_2_-0.2 M ascorbate buffer) [24]. After concentration, DNA was extracted from each concentrate (500 µL) via the AllPrep DNA/RNA Mini Kit (Qiagen, CA, USA). DNA libraries were prepared using the modified Nextera XT protocol. Briefly, each of the viral DNA extracts were used in a tagmentation reaction, followed by 13 cycles of PCR with the Nextera i7 and i5 index primers and 2× Kapa master mix, and then sequenced on the Illumina HiSeq 4000 platform (Illumina, San Diego, CA, USA).

#### Metagenome assembly and analysis

Viromes were assembled and assigned taxonomy/function as described previously [25]. Briefly, paired-end reads were trimmed, merged, and *de novo* assembled using Trimmomatic ver. 0.36 (slidingwindow:4:30 minlen:60) [26], FLASh ver. 1.2.11 [27], and metaSPAdes ver. 3.10.1 (without read error correction), respectively [28]. MetaGene was then used to predict open reading frames (ORFs) from each assembly [29]. Peptide sequences encoded by the predicted ORFs were queried against the peptide SEED and Phage SEED databases (retrieved 11/17/2017) using protein–protein BLAST (BLASTp ver. 2.6.0+) (E value ≤ 1e^−3^) [30, 31].

Coverage was calculated for each contig by: (i) recruiting quality-controlled reads to contigs via Bowtie2 ver. 2.3.3 (very sensitive local mode) (ii) processing the BAM file for artificial duplicates using Picard (https://broadinstitute.github.io/picard/), and then (iii) using the “depth” function of Samtools ver. 1.4.1 to compute the per-contig coverage [32, 33]. ORF coverage was denoted by start and stop coordinates within each contig. To normalize the abundances, contig and ORF coverages were divided by the sum of coverage per million, similar to the transcripts per million (TPM) metric used in RNA-Seq [34]. Assignment data were visualized using ggplot2 and heatplus [35, 36].

### Results

#### VLP abundance

**Fig. 1:**
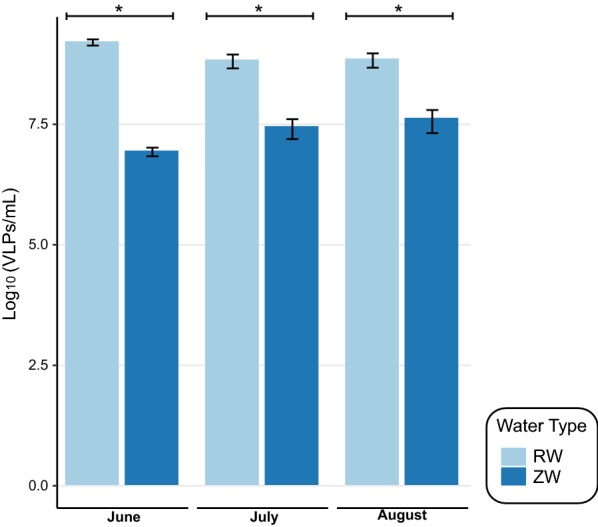
Epifluorescent microscopy counts of virus-like-particles (VLPs) in reclaimed water (RW) and zero-valent iron sand filtered reclaimed water (ZW). Samples were collected monthly from June through August. Data presented as mean ± SD, denoted by error bars. Significance determined relative to unfiltered reclaimed water at corresponding sampling dates (*p < 0.05)

At each sampling date, the VLPs were significantly (*p* < 0.05) less abundant in the ZW samples compared to the RW samples (Fig. 1). RW samples contained an average of 1.6 × 10^9^, 6.7 × 10^8^, and 7.0 × 10^8^ VLPs/mL in June, July, and August, respectively. The ZW samples contained an average of 8.6 × 10^6^, 2.8 × 10^7^, and 4.2 × 10^7^ VLPs/mL in June, July, and August, respectively.

#### Sequencing effort and assembly

Viral DNA was extracted from the six samples; however, it was not possible to obtain enough DNA from the June ZW sample for shotgun sequencing. From the samples that were sequenced, there were a total of 136,267,357 read pairs (Additional file 1: Table S1), with an average of 27,253,471 read pairs per virome (± 3,234,104 SD). Metagenomic assembly produced a total of 825,658 contigs, with an average of 165,132 contigs (± 30,305 SD) and an average of 278,196 ORFs (± 63,500 SD) per virome.

#### ORF clusters

To assess the percentage of functional similarity between RW and ZW viromes, peptide ORFs originating from the same sampling dates (July and August) were clustered using CD-HIT (60% peptide similarity) [37]. In July, 42% of the RW peptide ORFs clustered with 68% of the ZW peptide ORFs. For August, the percentage of shared function increased for the reclaimed sample; 60% of the RW peptide ORFs clustered with 61% of the ZW peptide ORFs (Additional file 2: Fig. S1).

#### Taxonomic assignment

**Fig. 2:**
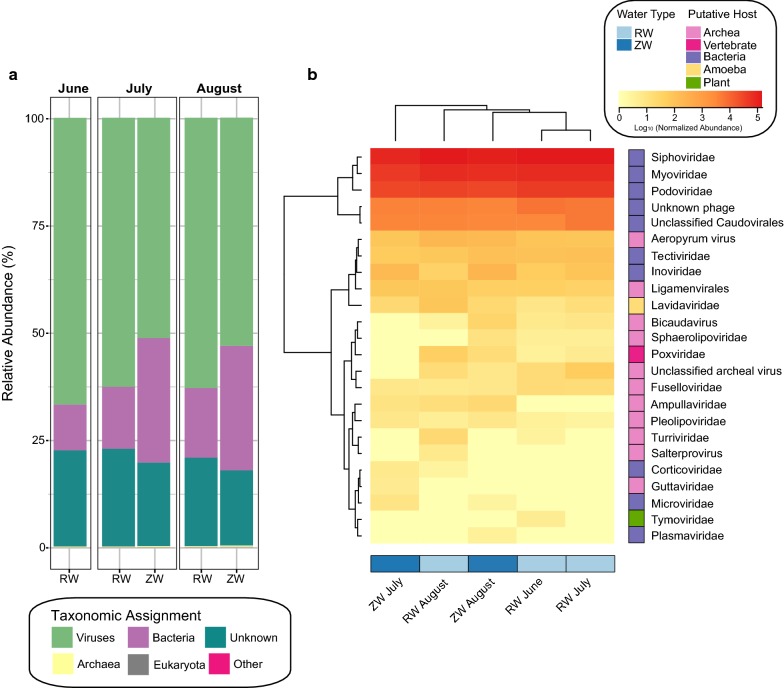
Taxonomic composition of reclaimed water (RW) and zero-valent iron sand filtered reclaimed water (ZW). **a** Percent of the normalized abundances (relative abundance) of the taxonomic assignments for RW and ZW viromes. **b** Heatmap showing the normalized abundances (logx + 1 transformed) of the viral taxa in the RW and ZW viromes. The heatmap has samples as columns (colored by water type) and viral taxa as rows (colored by putative host). Normalized abundance measured as contig coverage divided by the sum contig coverage per million

Similar to other virome studies [12], between 32–38% of contigs could be assigned a taxonomic representative (Additional file 1: Table S2**)**. For both the RW and ZW viromes, viral phyla (51–67%) accounted for the majority of the normalized abundance of the taxonomically assigned contigs, followed by bacteria (11–29%) and unknown (17–23%). However, the proportion of bacteria-assigned contigs was greater in the ZW viromes ( ~ 29%) than the RW (11–17%) (Fig. 2a).

The most abundant viral taxonomic classifications for each virome ( ~ 98% of all viral classified taxa) belonged to the dsDNA phages of the order *Caudovirales *(Fig. 2b), largely *Siphoviridae* (51–54%), followed by *Myoviridae* (28–31%), and *Podoviridae* (13–16%). The remaining ~ 2% of viral sequences were assigned as unclassified phage and viruses with putative hosts archaea, amoeba, plants, or vertebrates.

#### Functional assignment

**Fig. 3:**
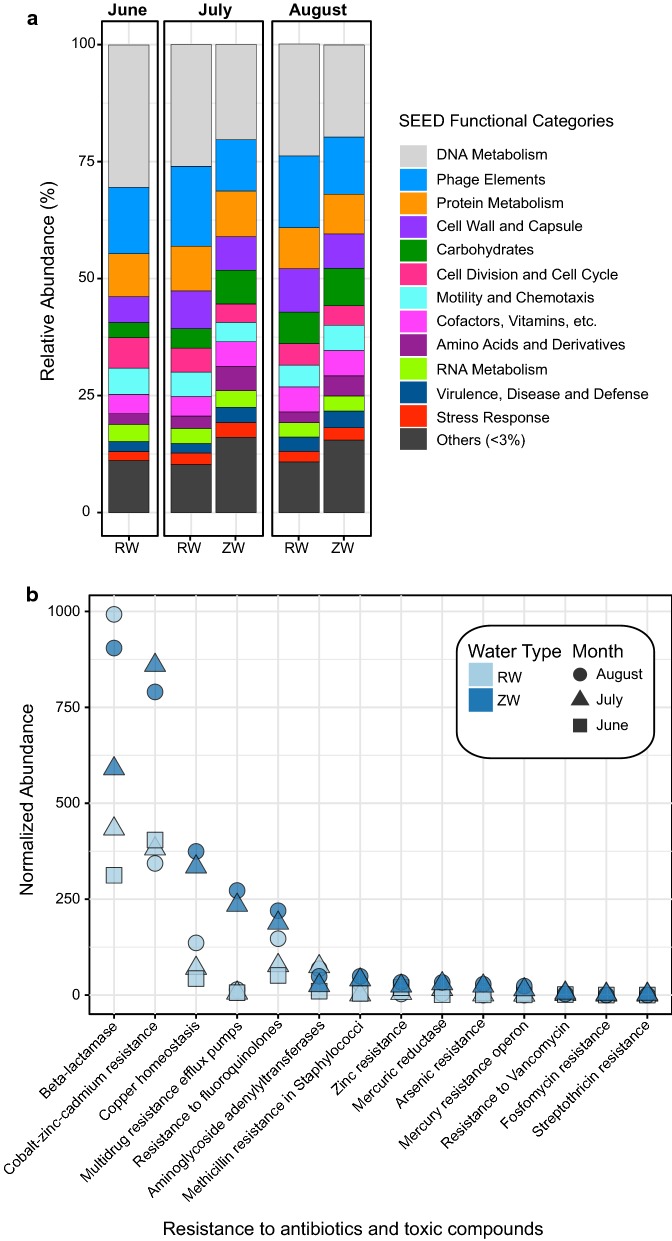
Functional composition of reclaimed water (RW) and zero-valent iron sand filtered reclaimed water (ZW). **a** Percent of the normalized abundances (relative abundance) of the SEED subsystems assignments for RW and ZW viromes. **b** Normalized abundance of antibiotic resistance genes in RW and ZW viromes. Shapes denote month samples were collected (June, square; July, triangle; August, circle) and color denotes water type (RW, light blue; ZW, dark blue). Normalized abundance measured as ORF coverage divided by sum ORF coverage per million

For both sample types the SEED Subsystem, DNA metabolism, was the most abundant, accounting for 20–30% of the total normalized abundance assigned to functionally classified peptide ORFs. This was followed by phage elements (11–17%), and protein metabolism (8–10%) (Fig. 3a). Annotated SEED Subsystem assignments were parsed for those assigned as resistant to antibiotics and toxic compounds, which were only between 1 and 2%. Among the putative antibiotic and toxic compound annotations, genes for Beta-lactamase were dominant in both of the August viromes. Additionally, the ZW viromes had a greater normalized abundance than all of the RW viromes for: cobalt-zinc-cadmium resistance, copper homeostasis, multidrug resistance efflux pumps, fluoroquinolone resistance, methicillin resistance in staphylococci, zinc resistance, mercuric reductase, arsenic resistance, and mercury resistance operon (Fig. 3b).

### Discussion

Reclaimed water is an important emerging resource that can help alleviate stress on freshwater systems [38, 39]. However, concerns remain regarding microbiological and chemical constituents persisting in reclaimed water, and whether treatment technologies can be used for further remediation. Here, we found that reclaimed water harbors 10^8^–10^9^ VLPs/mL, comparable to the abundances published in previous similar studies [12, 40]. After ZVI-sand filtration, the number of VLPs was significantly lower at all dates, ranging between 10^6^ and 10^7^ VLPs/mL, a roughly 1–2 log reduction. Previous studies have suggested that virus removal from water during ZVI-sand filtration is likely attributed to adsorption and inactivation via iron oxides within the iron [6, 41]. You et al. suggested that as water flows through a ZVI-sand filter, new iron oxides are formed continuously, generating additional adsorption sites that can extend the life of the filter [6].

Our findings are similar to recent results on the reduction efficiency of sand filtration alone for viruses ϕX174, MS2, and AiV ( < 1–2 log) and lower than previous studies on ZVI-sand filtration, which reported that φX174 and MS-2 were reduced by 4–6 logs [6, 7]. However, these studies focused largely on the removal of a few specific viral species and, even so, have found that removal efficiencies vary among species [7]. Here, we used epifluorescence microscopy to look at the entire viral population. This includes hundreds to thousands of different species, with a range of capsid sizes and isoelectric points, which may explain the smaller removal efficiency [42]. Moreover, while the log-reduction is lower than expected for the overall population, the total VLP concentration post ZVI-sand filtration is still comparable to those described in well and potable water [12, 43].

In terms of viral taxonomic composition, both RW and ZW viromes were dominated by *Siphoviridae*, which are abundant in human waste and reclaimed water [12, 44]. These viruses present a unique risk, as the majority of cultured representatives are capable of lysogeny and, thus, may facilitate horizontal gene transfer among bacteria [45]. Additionally, in both sample types the functional profiles were largely composed of genes related to DNA metabolism (Fig. 3a). A previous study of wastewater also found these genes to be highly abundant and attributed this to the elevated metabolic activity within wastewater treatment plants [40].

In addition, ZW viromes had a greater relative abundance of bacterial assigned contigs. In virome studies, sequences with significant homology to bacteria are sometimes unknown prophage embedded in a bacterial genome present in the database, or phages carrying host genes [12, 46]. During use, ZVI produces ROS, which can promote prophage induction [47, 48]. Thus, during filtration, ROS may stimulate the induction of integrated prophages. However, while the abundance of some genes is higher in the ZW viromes, the overall number of gene copies is likely still higher in the RW sample due to the increased number of total VLPs. Therefore, additional work is necessary to determine whether the bacterial assigned contigs are indeed prophage and whether this impacts the dissemination of bacterial genes in water reuse systems.

## Limitations

Our study was limited by sample size (*n* = 6), which prevented a rigorous statistical analysis. In addition, we could not obtain enough DNA from the June ZW water sample for shotgun sequencing and, therefore, a comparison between RW and ZW water for June was not possible. Finally, because we were not able to include a second sand-only filter control—due to the set-up of the larger study—we were unable to tease out the effects of ZVI versus sand in terms of virus removal.

## Additional files

**Additional file 1: Table S1.** Sequencing statistics for viral metagenomic samples.** Table S2.** Viral metagenomic contigs assigned taxonomy.

**Additional file 2: Figure S1.** ORF clustering in paired reclaimed water (RW) and ZVI sand filtered reclaimed water (ZW) samples from July and August.

## Abbreviations

RW: reclaimed water
ZVI: zero-valent iron
ZW: zero-valent iron filtered reclaimed water
ORF: open reading frame
VLP: virus like particle
