# Supplementary material for: Zero-valent iron sand filtration reduces concentrations of virus-like particles and modifies virome community composition in reclaimed water used for agricultural irrigation
Source: BMC Res Notes. 2019 Apr 11;12:223. doi: 10.1186/s13104-019-4251-y (PMC6458639; doi:10.1186/s13104-019-4251-y)
Supplement: Supplementary file 1 — Additional file 1: Table S1. Sequencing statistics for viral metagenomic samples. Table S2. Viral metagenomic contigs assigned taxonomy. [file 13104_2019_4251_MOESM1_ESM.docx]

**Table S1:** Sequencing statistics for viral metagenomic samples.

| Water Type | Sample Date | No. Read Pairs | No. Contigs | Avg. Contig Length | No. ORFs | %GC |
| --- | --- | --- | --- | --- | --- | --- |
| RW | 6/21/16 | 23,726,492 | 194,953 | 712 | 337,289 | 51.1 |
|  | 7/30/16 | 27,521,057 | 181,705 | 750 | 327,041 | 51.3 |
|  | 8/9/16 | 24,228,281 | 162,620 | 688 | 276,213 | 49.9 |
| ZW | 7/30/16 | 29,933,826 | 115,436 | 591 | 177,082 | 51.4 |
|  | 8/9/16 | 30,857,701 | 170,944 | 630 | 273,354 | 52.4 |

**Table S2:** Contigs assigned taxonomy.

| Water Type | Sample Date | % Assigned | Bacteria | Virus | Unknown | Archaea | Eukaroyota |
| --- | --- | --- | --- | --- | --- | --- | --- |
| RW | 6/21/16 | 32.8 | 8,698 | 39,377 | 15,656 | 193 | 26 |
|  | 7/30/16 | 32.6 | 9,221 | 35,074 | 14,804 | 165 | 19 |
|  | 8/9/16 | 32.8 | 10,029 | 30,711 | 12,308 | 181 | 69 |
| ZW | 7/30/16 | 35.8 | 15,669 | 17,519 | 7,936 | 137 | 61 |
|  | 8/9/16 | 38.2 | 24,713 | 28,839 | 11,410 | 244 | 91 |
