# Supplementary material for: Zero-valent iron sand filtration reduces concentrations of virus-like particles and modifies virome community composition in reclaimed water used for agricultural irrigation
Source: BMC Res Notes. 2019 Apr 11;12:223. doi: 10.1186/s13104-019-4251-y (PMC6458639; doi:10.1186/s13104-019-4251-y)
Supplement: Supplementary file 2 — Additional file 2: Figure S1. ORF clustering in paired reclaimed water (RW) and ZVI sand filtered reclaimed water (ZW) samples from July and August. [file 13104_2019_4251_MOESM2_ESM.pdf]

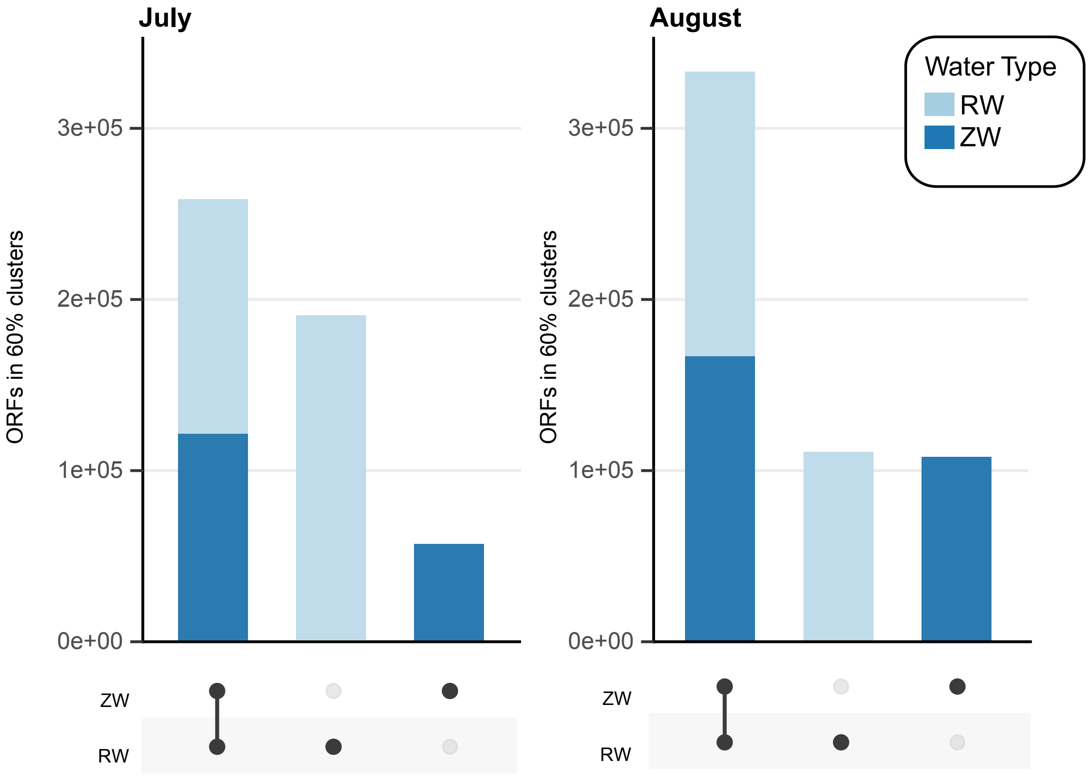

Figure S1: ORF clustering reveals unique and shared functional content in paired reclaimed water (RW) and ZVI sand filtered reclaimed water (ZW) samples from July and August. Bars denote the number of ORFs from each sample type contained within 60% similarity peptide clusters. Single bars depict the unique ORFs that clustered within water type, while stacked bars depict the ORFs that clustered between water types (e.g. shared ORFs).
